# Causal relationship between gut microbiota and urticaria: a bidirectional two-sample mendelian randomization study

**DOI:** 10.3389/fmicb.2023.1189484

**Published:** 2023-06-22

**Authors:** Yun-Zhou Shi, Qing-Feng Tao, Hai-Yan Qin, Ying Li, Hui Zheng

**Affiliations:** Acupuncture and Tuina School, Chengdu University of Traditional Chinese Medicine, Chengdu, China

**Keywords:** urticaria, gut microbiota, bidirectional, causal relationship, Mendelian randomization study

## Abstract

**Background:**

Cumulative evidence showed an association between gut microbiota and urticaria, but the causal relationship between them is unclear. We aimed to verify whether there is a causal relationship between the composition of gut microbiota and urticaria and explore whether the causal effect was bidirectional.

**Methods:**

We obtained genome-wide association studies (GWAS) summary data of 211 gut microbiota and urticaria from the most extensive available GWAS database. A bidirectional two-sample mendelian randomization (MR) study was used to test the causal relationship between the gut microbiota and urticaria. The MR analysis was primarily performed with the inverse variance weighted (IVW) method, and MR-Egger, weighted median (WM), and MR-PRESSO were performed as sensitivity analyses.

**Results:**

The Phylum Verrucomicrobia (OR 1.27, 95%CI 1.01 to 1.61; *p* = 0.04), Genus Defluviitaleaceae UCG011 (OR 1.29, 95%CI 1.04 to 1.59; *p* = 0.02), and Genus Coprococcus 3 (OR 1.44, 95%CI 1.02 to 2.05; *p* = 0.04) was a risk effect against urticaria. And Order Burkholderiales (OR 0.68, 95%CI 0.49 to 0.99; *p* = 0.04) and Genus *Eubacterium xylanophilum* group (OR 0.78, 95%CI 0.62 to 0.99; *p* = 0.04) were negatively associated with urticaria, suggesting a protective effect. At the same time, urticaria had a positively causal effect on gut microbiota (Genus *Eubacterium coprostanoligenes* group) (OR 1.08, 95%CI 1.01 to 1.16; *p* = 0.02). These findings showed no influence by heterogeneity or horizontal pleiotropy. Moreover, most sensitivity analyses showed results consistent with those of IVW analysis.

**Conclusion:**

Our MR study confirmed the potential causal relationship between gut microbiota and urticaria, and the causal effect was bidirectional. Nevertheless, these findings warrant further examination owing to the unclear mechanisms.

## 1. Introduction

Urticaria is a skin disease marked by the appearance of wheals (hives), angioedema, or both. When a person has transient wheals that last more than 6 weeks and occur virtually daily, it is defined as chronic urticaria (CU), chronic spontaneous urticaria (CSU) is the most common form of CU, characterized by recurrent itchy wheals and/or angioedema lasting for more than 6 weeks without any specific eliciting factors (Schaefer, 2017; Zuberbier et al., 2022). Its incidence had reportedly increased exponentially in the past few years (Gonçalo et al., 2021). About 1% of the world population, primarily young and middle-aged women, were affected by urticaria (Fricke et al., 2020). According to the relative evidence, urticaria can cause anxiety, depression, sleep, sexuality disturbances, and severely impair quality of life (Arias-Cruz et al., 2018; Choi G.-S. et al., 2020; Sánchez-Díaz et al., 2022). Besides, the cumulative evidence showed that patients with CSU can experience a significant loss of productivity at work, school, or in daily activities (Balp et al., 2017, 2018; Itakura et al., 2018). Moreover, the decline in productivity also resulted in high direct and indirect healthcare costs to treat urticaria, with significant socioeconomic impacts (Graham et al., 2016; Parisi et al., 2016).

Existing research showed that gut microbiota played a vital role in the health and disease of the host (Yao et al., 2022). The gut-skin axis was a relatively recent concept that referred to the bidirectional relationship between the gut microbiome and skin. Increasing evidence suggested that changes in the gut microbiome can trigger skin inflammation (Kim et al., 2020; Chun et al., 2021). It was worth noting that the 16S ribosomal RNA gene sequencing results showed that microbial composition was significantly different between urticaria patients and healthy individuals (Lu et al., 2019; Ćesić et al., 2021; Wang et al., 2021; Zhang et al., 2021). Besides, a randomized placebo-controlled trial suggested adjunct therapy with probiotics was safe and effective at four weeks in treating chronic urticaria in children (Bi et al., 2021). Although cumulative evidence suggested a correlation between gut microbiota composition and urticaria, it is still unclear whether particular gut microbiota taxa cause urticaria or urticaria leads to changes in gut microbiota. The above studies focusing on gut microbiota diversity cannot make a causal inference.

**Figure 1:**
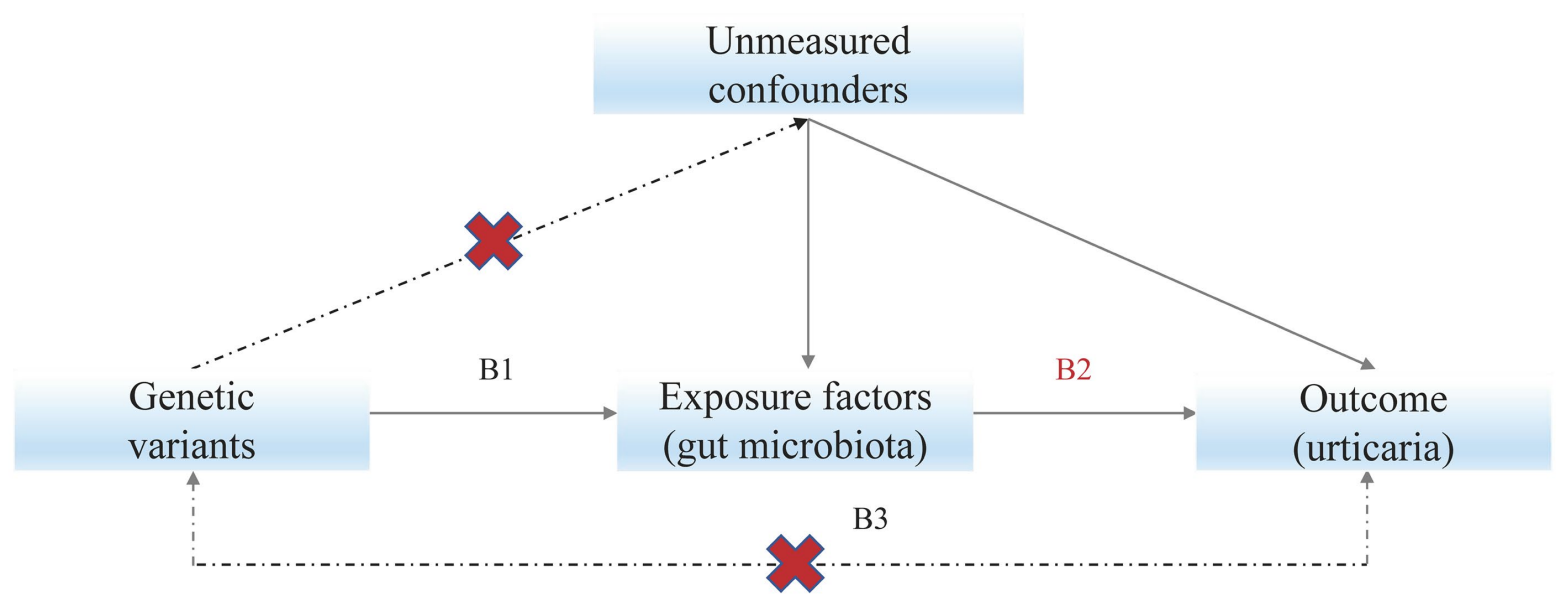
Mendelian randomization model. Subscript: Solid paths are theorized to exist; dashed paths are theorized to be nonsignificant according to Mendelian randomization assumptions; B2 indicates the causal relationship of interest to be estimated, where B2 = B1/B3. B1 and B3 represent the estimated direct effects of a genetic variant on the exposure (e.g., gut microbiota) and outcome (e.g., urticaria), respectively.

Mendelian randomization (MR) studies use genetic instruments, normally single nucleotide polymorphisms (SNPs), to detect the causal effects of exposures on outcomes (Figure 1). The MR studies are analogous to randomized controlled trials as there is an equal probability of either allele being randomly inherited by an individual (Emdin et al., 2017). Compared with observational studies like case–control studies, MR studies are less affected by confounding issues and can make causal inferences (Bowden and Holmes, 2019).

Therefore, based on the above findings and the MR method, we aimed to conduct an MR study first to verify the causal effect of gut microbiota taxa on the pathogenesis of urticaria. We then examined whether the causal effect was bidirectional.

## 2. Methods

### 2.1. Study design

We utilized a bidirectional two-sample MR to assess the causal association between gut microbiota and urticaria, using summary-level data from the publicly available Genome-Wide Association Study (GWAS). Data from the GWAS study were obtained from UK Biobank. The study design and reporting conformed to STROBE-MR (Skrivankova et al., 2021).

### 2.2. Ethics statement

Our analyses used published studies or publicly available GWAS abstract data, and therefore did not require ethics committee approval. Each study included was approved by its institutional ethics review board.

### 2.3. Data sources

The gut microbiota and urticaria were selected as exposure or outcome variables for this study of MR, respectively. The instrumental variables used for the endpoints came from the UK Biobank. UK Biobank study offers an excellent opportunity to identify novel genetic variants affecting gut microbiota and help examine causal relationships between gut microbiota and urticaria. Due to strict inclusion and exclusion criteria, single nucleotide polymorphisms (SNPs) significantly associated with diverse gut microbiota were selected as instrumental variables (IVs). A series of sensitivity analyses were performed for significant associations. In addition, we performed a reverse MR study to observe the bidirectional causal effect of urticaria on gut microbiota, using urticaria as the exposure variable and gut microbiota as the outcome variable.

### 2.4. Instrumental variables selection

MR studies can be used for unbiased estimation of causal effects, and the studies of genetic variation require three conditions to be met (Sekula et al., 2016):

1. The variant loci used as an instrumental variable (IV) must be related to the exposure factor (gut microbiota) to be studied;
2. The variant loci cannot be associated with any confounding factors;
3. The effect of instrumental variables on the outcome variable production exists directly through the exposure factor and independently of other factors.

We used the following selection criteria for genetic instruments: ① SNPs significantly associated with gut microbiota were selected as instrumental variables. One threshold value was used to select the instrumental variable. Since no SNPs were associated with gut microbiota up to a genome-wide significance threshold [*p* < 5 × 10^−8^], a significance level [*p* < 1 × 10^−6^] is recommended to extract instrumental variables; ② One of the principles of the MR approach is that there is no linkage disequilibrium (LD) among the included instrumental variables since strong LD might result in biased results. In the current study, a clustering process (*R*^2^ < 0.001, clustering distance: KB = 10,000) was performed to assess the LD between the included SNPs to remove the LD; ③ To assess whether any genetic tools have been previously associated with urticaria and to remove possible confounders, we searched the PhenoScanner GWAS database (PhenoScanner (cam.ac.uk)). If relevant phenotypes had been previously reported as urticaria risk factors, we would exclude those risk factors to remove possible confounders, satisfying hypothesis 2 of the MR approach. The phenotypes of gut microbiota-related SNPs were similarly searched in the PhenoScanner GWAS database, and SNPs whose phenotypes were consistent with urticaria were excluded, satisfying hypothesis 3; ④ A vital step of MR is to ensure that the effects of the SNPs on the exposure correspond to the same allele as the effects on the outcome. Echo SNPs would not be included in the instrumental variables following the principle. Finally, RStudio synthesized the dataset.

### 2.5. Statistical analysis

We coordinated SNP-gut microbiota and SNP-urticaria data and performed a two-sample MR analysis using the TwosampleMR R package (version 4.1.1). Since all genetic variants are unlikely to be valid instrumental variables, several robust approaches have been proposed. The method of Inverse variance weighted (IVW Method), Method of Weighted-Median (Weighted-Median Method), and Method of Egger regression (MR-Egger Method) were used in the preliminary analysis to estimate the impact. The MR-Egger intercept is a method with the property that detects and adjusts pleiotropy in the MR analysis and gets a causal effect estimate (Bowden et al., 2016). It examines whether the results are driven by the directional horizontal pleiotropy (Burgess and Thompson, 2017). We used the IVW method and MR-Egger regression to detect heterogeneity. The heterogeneities were quantified by Cochran *Q* statistic, a *p*-value<0.05 would be considered significant heterogeneity (Wu et al., 2020). Given the lower accuracy and statistical power of MR-Egger regression, MR pleiotropy residual sum and outlier (MR-PRESSO) was performed to detect any outliers reflecting likely pleiotropic biases and correct horizontal pleiotropy (Xiang et al., 2021).

## 3. Results

### 3.1. Results of the causal effect of gut microbiota on urticaria

**Figure 2:**
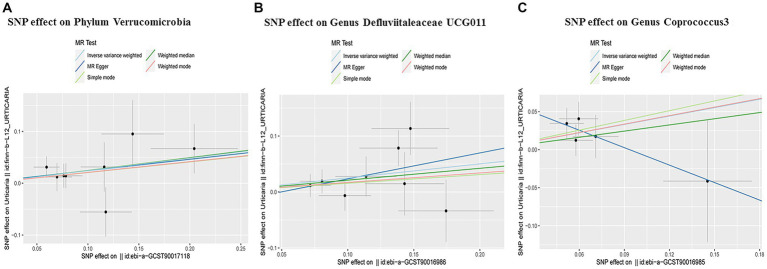
Scatter plots of three taxa of gut microbiota positively associated with urticaria. Subscript: scatter plots of the taxa-SNP associations (x-axis) versus the urticaria-SNP associations (y-axis) were shown, with horizontal and vertical lines showing 95% confidence intervals for each association. The MR analysis was performed primarily with the inverse variance weighted method and secondarily examined with the MR-Egger, the weighted median, and other methods. The lines that move obliquely upward from left to right show a positive correlation of the taxa with urticaria, indicating a pathogenic causal effect. The lines that are inclined down indicate a protective causal effect. MR, mendelian randomization. SNP, single nucleotide polymorphisms.

We found that the Phylum Verrucomicrobia was positively associated with urticaria, suggesting a risk effect of the Phylum Verrucomicrobia on urticaria (OR 1.27, 95%CI 1.01 to 1.61; *p* = 0.04; Table 1; Figure 2A) in the IVW analysis. Maximum likelihood (OR 1.28, 95% CI 1.01 to 1.62; *p* = 0.04) and IVW radial (OR 1.27, 95%CI 1.05 to 1.55; *p* = 0.01) and IVW (fixed effects) (OR 1.27, 95%CI 1.01 to 1.61; *p* = 0.04) confirmed the finding. However, the MR-Egger and Weighted median analysis did not support the result. Furthermore, the result suggested no directional horizontal pleiotropy (Egger intercept = 0.002; *p* = 0.94). MR-PRESSO analysis showed no evidence of horizontal pleiotropy (Residual sum of squares 5.93; *p* = 0.73) (Table 2). The Cochran’s Q test showed no evidence of heterogeneity (Cochran’s Q = 4.86; *p* = 0.68) (Table 1).

**Table 1:** Results of MR analysis of the causal effect of gut microbiota on urticaria using different analytical methods.

Exposure	N.SNPs	Methods	Beta	SE	OR (95% CI)	*p* value	Cochran’s Q Statistic	Heterogeneity *p*-value
Phylum Verrucomicrobia	8	IVW	0.24	0.12	1.27 (1.01,1.61)	0.04	4.86	0.68
		MR-Egger	0.22	0.32	1.25 (0.67,2.32)	0.51	4.86	0.56
		weighted median	0.25	0.15	1.28 (0.94,1.74)	0.10	NA	NA
		Maximum Likelihood	0.25	0.12	1.28 (1.01,162)	0.04	NA	NA
		IVW radial	0.24	0.10	1.27 (1.05,1.55)	0.01	NA	NA
		IVW (fixed effects)	0.24	0.12	1.27 (1.01,1.61)	0.04	NA	NA
Genus Defluviitaleaceae UCG011	8	IVW	0.25	0.11	1.29 (1.04,1.59)	0.02	7.93	0.34
		MR-Egger	0.46	0.40	1.59 (0.72,3.52)	0.30	7.55	0.27
		Weighted median	0.21	0.14	1.23 (0.94,1.63)	0.15	NA	NA
		Maximum Likelihood	0.27	0.11	1.31 (1.06,162)	0.01	NA	NA
		IVW radial	0.25	0.11	1.28 (1.04,1.59)	0.02	NA	NA
		IVW (fixed effects)	0.25	0.10	1.29 (1.05,1.57)	0.01	NA	NA
Genus Coprococcus3	5	IVW	0.37	0.18	1.44 (1.02,2.05)	0.04	2.96	0.56
		MR-Egger	−0.77	0.83	0.47 (0.09,2.35)	0.42	0.99	0.80
		Weighted median	0.27	0.23	1.31 (0.84,2.05)	0.24	NA	NA
		Maximum Likelihood	0.37	0.18	1.45 (1.01,2.08)	0.04	NA	NA
		IVW radial	NA	NA	NA	NA	NA	NA
		IVW (fixed effects)	0.37	0.18	1.44 (1.02,2.05)	0.04	NA	NA
Order Burkholderiales	8	IVW	−0.39	0.19	0.68 (0.46,0.99)	0.04	13.78	0.06
		MR-Egger	−0.37	0.76	0.68 (0.16,3.05)	0.64	13.78	0.03
		Weighted median	−0.34	0.21	0.71 (0.47,1.07)	0.10	NA	NA
		Maximum Likelihood	−0.42	0.15	0.65 (0.50,0.88)	0.00	NA	NA
		IVW radial	−0.39	0.19	0.68 (0.46,0.99)	0.04	NA	NA
		IVW (fixed effects)	−0.39	0.14	0.68 (0.52,0.89)	0.00	NA	NA
Genus *Eubacterium xylanophilum* group	7	IVW	−0.25	0.12	0.78 (0.62,0.99)	0.04	4.18	0.65
		MR-Egger	−0.35	0.36	0.70 (0.35,1.41)	0.37	4.08	0.54
		Weighted median	−0.34	0.16	0.71 (0.52,0.96)	0.03	NA	NA
		Maximum Likelihood	−0.25	0.12	0.78 (0.61,0.99)	0.04	NA	NA
		IVW radial	−0.25	0.10	0.78 (0.64,0.95)	0.01	NA	NA
		IVW (fixed effects)	−0.25	0.12	0.78 (0.62,0.99)	0.04	NA	NA

MR, Mendelian randomization; IVW, inverse-variance weighted; SNP, single nucleotide polymorphism; Beta, beta coefficient; N.SNPs, number of SNPs used in MR; OR, odds ratio; 95%CI, confidence interval; SE, standard error.

**Table 2:** MR-PRESSO estimates gut microbiota on urticaria.

Traits	Raw estimates			Outlier-corrected estimates			Residual sum of squares	*p*-value
	N	SD	*p*-value	N	SD	*p*-value		
Phylum Verrucomicrobia	8	0.10	0.04	8	NA	NA	5.93	0.73
Genus Defluviitaleaceae UCG011	8	0.11	0.06	8	NA	NA	10.56	0.35
Genus Coprococcus 3	5	0.16	0.18	5	NA	NA	7.36	0.46
Order Burkholderiales	8	0.17	0.12	8	NA	NA	20.28	0.06
Genus *Eubacterium xylanophilum* group	7	0.10	0.08	7	NA	NA	7.40	0.60

The Genus Defluviitaleaceae UCG011 was also found to be risk against urticaria (OR 1.29, 95%CI 1.04 to 1.59; *p* = 0.02; Table 1; Figure 2B) in the IVW analysis. Maximum likelihood (OR 1.31, 95%CI 1.06 to 1.62; *p* = 0.01) and IVW radial (OR 1.28, 95%CI 1.04 to 1.59; *p* = 0.02) and IVW (fixed effects) (OR 1.29, 95%CI 1.05 to 1.57; *p* = 0.01) confirmed the finding. However, the MR-Egger and Weighted median analysis did not support the result. Moreover, the result also suggested no directional horizontal pleiotropy (Egger intercept = −0.02; *p* = 0.60). MR-PRESSO analysis showed no evidence of horizontal pleiotropy (Residual sum of squares = 10.56; *p* = 0.35) (Table 2). The Cochran’s Q test showed no evidence of heterogeneity (Cochran’s Q = 7.93; *p* = 0.34) (Table 1).

At the same time, the Genus Coprococcus 3 was also positively associated with urticaria. The IVW analysis showed that the Genus Coprococcus 3 was a risk effect for urticaria (OR 1.44, 95%CI 1.02 to 2.05; *p* = 0.04; Table 1; Figure 2C). The result suggested no directional horizontal pleiotropy (Egger intercept = 0.07, *p* = 0.26). MR-PRESSO analysis showed no evidence of horizontal pleiotropy (Residual sum of squares = 7.36; *p* = 0.46) (Table 2). The Cochran’s Q test showed no evidence of heterogeneity (Cochran’s Q = 2.96, *p* = 0.56) (Table 1).

**Figure 3:**
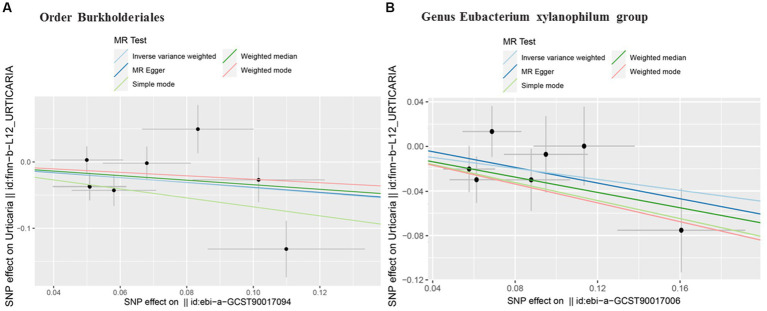
Scatter plots of two taxa of gut microbiota negatively associated with urticaria. Subscript: scatter plots of the taxa-SNP associations (x-axis) versus the urticaria-SNP associations (y-axis) were shown, with horizontal and vertical lines showing 95% confidence intervals for each association. The MR analysis was performed primarily with the inverse variance weighted method and secondarily examined with the MR-Egger, the weighted median, and other methods. The lines that move obliquely upward from left to right show a positive correlation of the taxa with urticaria, indicating a pathogenic causal effect. The lines that are inclined down indicate a protective causal effect. MR, mendelian randomization. SNP, single nucleotide polymorphisms.

Interestingly, we found that gut microbiota is also a protective factor for urticaria. In the Order Burkholderiales (OR 0.68, 95%CI 0.46 to 0.99; p = 0.04; Table 1; Figure 3A) and Genus *Eubacterium xylanophilum* group (OR 0.78, 95%CI 0.62 to 0.99; p = 0.04; Table 1; Figure 3B) was negatively associated with urticaria in the IVW method analysis. The other different analytical methods, except for MR Egger’s analysis showed similar results (Table 1). Sensitivity analysis (Table 2) showed no potential horizontal pleiotropy. Cochran’s Q test indicated no significant heterogeneity (Table 1).

### 3.2. Results of the causal effect of urticaria on gut microbiota

**Figure 4:**
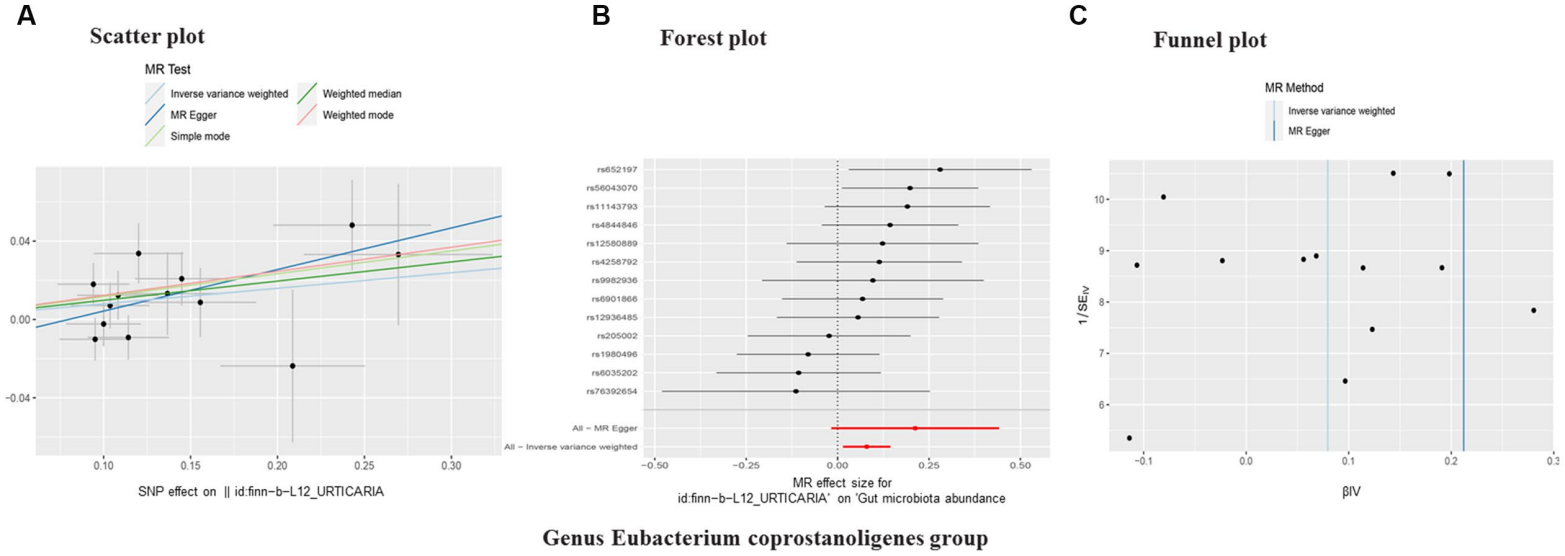
Scatter plot **(A)**, forest plot **(B)**, funnel plot **(C)** of the causal effect of urticaria on gut microbiota (Genus *Eubacterium coprostanoligenes* group). Subscript: In a scatter plot, the slope of the line indicates a pathogenic cause-effect relationship. Forest plot showing the causal effect of urticaria on gut microbiota. Funnel plot showing the overall heterogeneity of MR estimates of urticaria on gut microbiota. MR, mendelian randomization. SNP, single nucleotide polymorphisms.

In reverse causality, urticaria is the exposure factor used to demonstrate gut microbiota results. Again, we used a random effects model. According to the IVW method, urticaria had a positive causal effect on the gut microbiota (Genus *Eubacterium coprostanoligenes* group) in our study (OR 1.08, 95%CI 1.01 to 1.16; *p* = 0.02; Table 3; Figure 4). And other different analytical methods also confirmed the finding except for MR-Egger analysis (Table 3). The result suggested no directional horizontal pleiotropy (Egger intercept = −0.02; *p* = 0.26). MR-PRESSO analysis showed no evidence of horizontal pleiotropy (Residual sum of squares = 20.42; *p* = 0.30). The Cochran’s Q test showed no evidence of heterogeneity (Cochran’s Q = 12.78; *p* = 0.39) (Table 3).

**Table 3:** Results of MR analysis of the positive causal effect of urticaria on gut microbiota using different analytical methods.

Exposure	N.SNPs	Methods	Beta	SE	OR (95% CI)	*p* value	Cochran’s Q Statistic	Heterogeneity *p*-value
Genus *Eubacterium coprostanoligenes* group	13	IVW	0.08	0.03	1.08 (1.01,1.16)	0.02	12.78	0.39
		MR-Egger	0.21	0.12	1.24 (0.98,1.55)	0.10	11.35	0.41
		Weighted median	0.10	0.04	1.10 (1.01,1.21)	0.04	NA	NA
		Maximum Likelihood	0.08	0.03	1.09 (1.02,1.16)	0.01	NA	NA
		IVW radial	0.08	0.03	1.08 (1.01,1.16)	0.02	NA	NA
		IVW (fixed effects)	0.08	0.03	1.08 (1.01,1.15)	0.01	NA	NA

MR, Mendelian randomization; IVW, inverse-variance weighted; SNP, single nucleotide polymorphism; Beta, beta coefficient; N.SNPs, number of SNPs used in MR; OR, odds ratio; 95%CI, confidence interval; SE, standard error.

## 4. Discussion

### 4.1. Main findings

We performed a bidirectional two-sample MR analysis of the potential causal relationship between gut microbiota and urticaria using publicly available pooled data from GWAS, and our analysis supported a causal association between gut microbiota and urticaria. We found that the Phylum Verrucomicrobia, Genus Defluviitaleaceae UCG011, and Genus Coprococcus 3 was a risk effect against urticaria. And Order Burkholderiales and Genus *Eubacterium xylanophilum* group was negatively associated with urticaria, suggesting a protective effect. At the same time, urticaria had a positive causal effect on gut microbiota (Genus *Eubacterium coprostanoligenes* group). These findings showed no influence by heterogeneity or horizontal pleiotropy. These results were examined through several sensitivity analyses—MR-Egger, weighted-median, MR-EXPRESSO, etc. Furthermore, most sensitivity analyses showed the results consistent with those of IVW analysis.

### 4.2. Interpretation of the results

As we all know, urticaria is one of the most common skin diseases with high incidence, but the pathogenesis is unclear. However, it has been shown that there is a strong and bidirectional correlation between digestive health and skin health, especially when it comes to homeostasis and allostasis of the skin (Duchnik et al., 2023). Both the skin and the intestine are active immune organs that are constantly exposed to the outside environment (Coates et al., 2019). Unbalanced microbiota diversity should be considered one of the most important underlying causes of allergic skin diseases (Kim et al., 2016). Altered intestinal microbial diversity (dysbiosis) increases host susceptibility, disrupts mucosal immune tolerance, and produces neurotransmitters directly or regulates the regulation of neurotransmitter metabolism pathways, further affecting skin health (Minciullo et al., 2014). Significantly, the increasing evidence showed that gut microbiota dysbiosis is associated with chronic skin inflammatory disorders (Marrs and Flohr, 2016; Petersen et al., 2019). At the same time, the cumulative evidence indicated a correlation between gut microbiota composition and urticaria (Lu et al., 2019; Ćesić et al., 2021; Wang et al., 2021; Zhang et al., 2021). They had confirmed that CSU differed from healthy people in the composition and function of the gut microbiome and the metabolome between intestinal microenvironment. The related research of the urticaria group versus the healthy control group about gut microbiota composition mainly focused on the intestinal microbiome diversity comparison, intestinal microbiome abundance comparison, and the characteristics of patients with urticaria gut flora metabolism function prediction. Most studies showed that there was no significant difference in the α-diversity of the gut microbiota between urticaria patients and the healthy population, and significant differences in β-diversity (Lu et al., 2019; Zhang et al., 2021), suggesting that the difference between groups was greater than the difference within groups. The dysregulation of microbiome in urticaria patients was confirmed. Furthermore, the specific differences of microbial communities at different levels were analyzed. At present, previous studies have primarily focused on changing relative amounts of a few common microbiotas between urticaria and microbiota (Nabizadeh et al., 2017; Su et al., 2021). Urticaria patients and healthy individuals showed some differences in the abundance of the phylum, order, family, genus and species of the gut microbiota. Firmicutes, Bacteroidetes, Proteobacteria, Verrucomicrobia and Actinobacteria were the major phyla of patients with urticaria gut microbiome (Lu et al., 2019; Wang et al., 2021; Zhang et al., 2021). However, there were some conflicting findings between different studies regarding trends in the abundance of intestinal flora. Similarly, our study had both the same findings and new results when compared to previous studies.

In this study, we confirmed the relationship and further identified that out-of-balance gut microflora can cause urticaria. According to a review of CU pathogenesis, the infection could be an essential trigger in CSU (Ensina et al., 2019). We found that Genus Coprococcus3, Genus Defluviitaleaceae UCG011 was a risk effect against urticaria. Coprococcus is a critical member of the Phylum Firmicutes and a core genus of intestinal bacteria. The previous research reported that Coprococcus played a vital role in immune responses (Xu et al., 2022) and correlated with the atopic disease severity (Nylund et al., 2015). Our study also found a relationship between Coprococcus and urticaria. Besides, we found, for the first time, that Defluviitaleaceae UCG011 was positively associated with urticaria. Previous studies have found a positive correlation between Defluviitaleaceae and intestinal tract inflammatory response (Zha et al., 2020), and a negative correlation with butyric acid levels (Bordoni et al., 2019), but the exact impact is still unclear. Our result also found that the Phylum Verrucomicrobia was positively associated with urticaria, which were consistent with the above research (Wang et al., 2021). But how it works is still unclear.

Interestingly, we found that Genus *Eubacterium xylanophilum* group, and Order Burkholderiales was negatively associated with urticaria, suggesting a protective effect for urticaria. Our result is consistent with Akram Rezazadeh et al. study (Rezazadeh et al., 2018), except for the type of gut microbiota. *Eubacterium xylanophilum* is a member of the Genus Eubacterium, which forms a part of the core human gut microbiome. It was reported that Genus Eubacterium played a critical role in energy homeostasis, colonic motility, immunomodulation, and gut inflammation suppression (Mukherjee et al., 2020). Similarly, Genus *Eubacterium xylanophilum* group was found to have a strong positive correlation with levels of proinflammatory cytokines (Gong et al., 2022), and have a linear correlation with radiation-induced intestinal injury (Zhao et al., 2021). This also indicated that intestinal inflammatory damage was closely related to bacterial imbalance. A randomized placebo-controlled trial showed that Adjunct therapy with probiotics for CU in children was safe and effective (Bi et al., 2021). This study provides new evidence for using probiotics as adjunctive therapy for urticaria. Some researchers also analyzed the roles of gut-microbiota and probiotics in urticaria (Liu et al., 2022). But the relative researchers had found that only case reports and uncontrolled studies existed on probiotics, and the global level of evidence was low or very low (Vena et al., 2017). Further high-quality studies are also needed to assess the effectiveness and safety of probiotics as an alternative therapy for urticaria. Besides, our MR study also showed that urticaria had a positive causal effect on gut microbiota. The previous studies had similar findings, showing that the microbial composition was significantly different between urticaria patients and the healthy individual (Lu et al., 2019; Wang et al., 2021).

It’s worth noting that there were some conflicting findings when compared to previous studies. We thought that a difference between our results and other studies may be partially due to differences in the sample size, geographical background, dietary habits, and age between the subjects in the different studies. The differences in microbiome composition may lead to dysfunction and abnormal metabolites. Accurate analysis of the intestinal microbiota may facilitate the establishment of an evaluating system.

To the best of our knowledge, our study is the first to use a two-sample bidirectional MR approach and various sensitivity analyses to investigate the causal relationship between gut microbiota and urticaria. We used the most extensive GWAS study to select genetic instruments, and our study was consistent with the correlation and independence hypothesis. In addition, We conducted a two-sample MR analysis, which can conduct a more extensive range of sensitivity analyses, yielding more robust estimates of causal effects (Choi Y. et al., 2020).

### 4.3. Limitation

Our study had several limitations. Firstly, the exposure and outcome studies used in two-sample MR analyses should not involve overlapping participants, whereas we may have overlapping participants in exposure and outcome studies, but it is not easy to estimate the extent of sample overlap. Bias from sample overlap must be minimized using robust instruments (e.g., *F*-statistics much larger than 10) (Pierce and Burgess, 2013). Secondly, we used a *p*-value<1 × 10^−6^ as a threshold, and the selection of a limited number of SNPs as IVs may explain only a tiny proportion of the variation in exposure, affecting the statistical power of causal estimates. Thirdly, The MR analysis was of European origin, and it remains to be verified whether the results represent the entire population. Fourthly, although we have confirmed a causal relationship between gut microbiota and urticaria, the mechanism of how specific gut microbiota works remains unclear and needs further study.

## 5. Conclusion

Our MR study confirmed the potential causal relationship between gut microbiota and urticaria, and the causal effect was bidirectional. But these findings warrant further examination owing to the unclear mechanisms.

## Data availability statement

The original contributions presented in the study are included in the article/Supplementary material, further inquiries can be directed to the corresponding author.

## Author contributions

Y-ZS designed the study and writing - original draft preparation. Y-ZS, Q-FT, and H-YQ done methodology, formal analysis, and data management. Y-ZS and HZ analyzed and interpreted the data. YL and HZ supervised and wrote and reviewed. Y-ZS wrote the first draft of the manuscript. All authors contributed to the article and approved the submitted version.

## Funding

This study was supported by the National Natural Science Foundation of China Youth Project (82105026), the 69th Batch of China Postdoctoral Foundation Project (2021 M693787), and the Sichuan Natural Science Foundation Project of China (23NSFSC2157).

## Conflict of interest

The authors declare that the research was conducted in the absence of any commercial or financial relationships that could be construed as a potential conflict of interest.

## Publisher’s note

All claims expressed in this article are solely those of the authors and do not necessarily represent those of their affiliated organizations, or those of the publisher, the editors and the reviewers. Any product that may be evaluated in this article, or claim that may be made by its manufacturer, is not guaranteed or endorsed by the publisher.
